# Familial Factors Associating with Youth Physical Activity Using a National Sample

**DOI:** 10.3390/children7070079

**Published:** 2020-07-15

**Authors:** Ryan D. Burns, Taylor E. Colotti, Christopher D. Pfledderer, You Fu, Yang Bai, Wonwoo Byun

**Affiliations:** 1 Department of Health and Kinesiology, University of Utah, Salt Lake City, UT 84108, USA; taylor.colotti@utah.edu (T.E.C.); chris.pfledderer@utah.edu (C.D.P.); Yang.Bai@utah.edu (Y.B.); won.byun@utah.edu (W.B.); 2 School of Community Health Sciences, University of Nevada, Reno, NV 89557, USA; youf@unr.edu

**Keywords:** adolescent, children, family, health, parents, physical activity, social support, survey

## Abstract

The purpose of this study was to examine the associations of familial and child-related factors with reported child physical activity using a representative sample of US children and adolescents. Data were analyzed from the combined 2017–2018 National Survey of Children’s Health. Household addresses were randomly selected within each US state. One parent within each household answered health and wellness questions pertaining to one randomly selected child (n = 37,392; 48.8% female; 6–17 years old). Weighted logistic regression models examined the independent and joint associations between family-level and child-level factors with a child meeting the 60 min of physical activity per day guideline. After controlling for confounders, higher levels of family resilience (odds ratio (OR) = 2.17; 95% confidence interval (CI): 1.35–3.49, *p* = 0.001), high event attendance (OR = 1.65; 95%CI: 1.18–2.31, *p* = 0.004), and high family income (OR = 1.84, 95%CI: 1.34–2.52, *p* < 0.001) significantly associated with higher odds of a child meeting the 60 min of physical activity per day guideline. Family generational status and adult education significantly modified the association between family resilience and child physical activity. Programs that develop family resilience and encourage parental attendance for their child’s events or activities may positively influence a child’s physical activity behaviors. Expanded or enhanced programming may be needed for lower income families.

## 1. Introduction

High levels of daily physical activity have numerous benefits for children and adolescents including a lower risk of cardiometabolic dysfunction, positive effects on cognitive functioning, and better emotional wellbeing [1,2,3]. Despite these benefits, many children and adolescents within the US and worldwide do not achieve the 60 min of physical activity per day guideline [4,5]. During childhood, numerous factors are thought to contribute to meeting physical activity guidelines, including individual determinants of behavior and determinants related to the social environment [6]. Because of this, researchers have suggested that the promotion of physical activity in children and adolescents should target multiple domains of influence [6]. Factors within the home may have a strong influence on child and adolescent behavior due to the higher relative amount of time a child spends within the home environment, specifically with parents and/or guardians. Previous studies have shown that parental social support for physical activity was positively correlated with child physical activity levels [7,8,9]. Previous studies have also shown that children tend to be more active if both parents support a child’s physical activity and sport endeavors [10].

The relationship between parent support and child physical activity aligns with socio-ecological models of health behavior that suggest that intraindividual and social environmental variables interact to promote physical activity [11]. Theoretical frameworks have also been proposed that link this reciprocal and dynamic relationship between parental engagement and support and child physical activity with child academic performance [12]. A meta-analysis of family-based physical activity interventions found significant and positive pooled effects for these interventions to increase physical activity within the pediatric population with a supplemental realist synthesis highlighting the importance of parental motivation for physical activity driving these pooled findings [13]. Other than perceived social support, the psychosocial variables of enjoyment and self-efficacy may drive the relationship between parent and child physical activity within dyads [14]. Furthermore, parents’ own sport activities and parents’ belief in sports’ capacities to foster personality development, character building, and social integration positively correlate with child physical activity [15,16]. Although many of these previous studies highlight the importance of parental support, they do not fully account for other familial factors that may confound the relationship, including the health of the parents [17], family generational status [18], parent educational levels [19], family poverty status [20], and the family structure [21], which all have shown to significantly correlate with child physical activity in previous work.

In addition to parental social support, a related familial factor that has also been shown to associate with health outcomes is family resilience. Resilience is the ability to withstand and rebound from crisis [22]. Adversity, crises, and life challenges that have an impact on the whole family manifest processes that lead to adaptations for individual family members, their interrelationships, and the family unit as a whole [22]. Higher levels of family resilience have been shown to correlate with child flourishing [23]. Families with higher levels of resilience associates with higher levels of parental social support and may yield more resilient children as they track through childhood and into adulthood [24]. Physical activity and physical literacy have been shown to correlate with child and adolescent resilience [25,26]. However, the direct link between family resilience and child physical activity is unknown.

Examining a comprehensive set of familial factors with child physical activity is important to inform researchers and practitioners that aim to derive interventions with a family component, which may yield higher levels of effectiveness for promoting child physical activity and its correlates [16,27]. Additionally, if specific familial factors such as family resilience and specific aspects of parental social support associates strongly with physical activity, schools and community centers have evidence to promote activities and events for both the children and their parents. Beets et al. [28], through a thorough literature review, identified four types of tangible parental social support that may facilitate child physical activity behaviors. One type of social support that was identified was “conditional-watching/supervision” which involves parents being present during child activities or events but not being actively involved. Parents who engaged in “conditional-watching/supervision” support tend to have a positive influence on the physical activity behaviors of their child [28]. Within the yearly administered National Survey for Children’s Health (NSCH), information is collected on the frequency of a child meeting the 60 min of daily physical activity guideline, family resilience, the frequency with which parents attend their child’s activities and/or events (conditional-watching/supervision support), in addition to the other important familial characteristics [29,30]. To the best of our knowledge, no study has examined the associations of family resilience, parental social support (conditional-watching/supervision), and child physical activity while also considering other important familial factors and important child-level covariates in a representative sample of US children and adolescents. Therefore, the purpose of this study was to examine the independent and joint associations between familial and child-level factors with reported weekly child physical activity using a representative sample of US children and adolescents.

## 2. Materials and Methods

### 2.1. Participants

Participants were a nationally representative sample of children and adolescents, aged 6–17 years old, whose parent completed either the 2017 or 2018 National Survey of Children’s Health (NSCH). The parents of the children provided all information that was utilized in the current study. The NSCH is a national survey that is fielded annually by the US Census Bureau and provides data on multiple aspects of children’s and adolescent’s physical, mental, and emotional health and wellbeing. The data for the 2017 and 2018 NSCH were combined with only items used in both datasets included in the current combined dataset [29,30,31]. The combined 2017–2018 NSCH provides opportunities for analyses using variables with small sample sizes or low prevalence. Physical activity data were only collected on children and adolescents aged 6–17 years old, therefore children younger than 6 years old were omitted from the current study. All direct identifiers, as well as characteristics that might lead to identification, had been omitted from the data files that were released to the public. NSCH data collection does not undergo an external Institutional Review Board review. Instead, the process for the review of methods and procedures was incorporated into the responsibilities of the US Census Bureau and Office of Management and Budget officials. This ensures that NSCH participant data were protected and treated with sensitivity [29,30,31]. The NSCH screener questionnaire indicated to the respondents that completion of the survey was voluntary and there would be no penalties for refusing to answer questions.

### 2.2. Procedures

Randomly selected addresses from households across the US were mailed instructions to access the NSCH online with some addresses also receiving a paper version of a screening questionnaire. Specifically, after two reminder letters and postcard reminders to complete the survey by web, those households who had not accessed the online survey were mailed a paper screening questionnaire. An indicator variable on the NSCH data release provided whether or not a respondent completed an online or paper questionnaire. An adult within the household was asked to complete the online or paper screener questionnaire to identify all children 0–17 years of age living in the household. If a child or children were reported to live in the household, adult participants were directed to a detailed age-specific topical questionnaire for one randomly selected child. Adult respondents completed one of three versions of the survey depending on the child’s age: 0–5 years, 6–11 years, 12–17 years. Screener questionnaires for the 2018 NSCH were mailed between June 2018 and January 2019 and the screener questionnaires for the 2017 NSCH were mailed between August 2017 and February 2018. Survey topics included child and family demographics, physical and mental health status, family health and activities, and parental health status, among others. To improve likelihood of response and reduce response bias, sampled addresses either received a USD 2 cash incentive (90% of the sample) or were part of a control group receiving a USD 0 incentive (10% of the sample). Based on web keystroke data and paper cognitive testing, the estimated survey length for households without children was 5 min and the estimated survey length for households with children was 39 min [29,30,31]. The latest NSCH data release (2018 NSCH) occurred in October 2019.

### 2.3. Data Processing

The 2017–2018 NSCH combined dataset contains two stratum identifiers corresponding to the state of residence and an identifier corresponding to households flagged with children [29]. The primary sampling unit was the unique household identifier. Because the statistical software program STATA only permits the specification of a single stratum variable, a new stratum variable was generated by combining the state of residence identifier with the identifier for households with children using STATA’s “group” function [29]. The 2017–2018 NSCH combined dataset also included an adjusted probability weight variable that accounted for combining two years of data [29,31]. Adjusted probability weights were obtained by applying a base weight to each household, adjusting for screener questionnaire nonresponse, followed by an eligible child adjustment to population controls, an adjustment for topical questionnaire non-response, and finally an adjustment made to match each state’s weighted survey response to selected characteristics of the state’s population of non-incarcerated children aged 0–17 years [29,30,31]. After survey weights were applied, extensive non-response bias analyses indicated no evidence for non-response bias on the 2017 and 2018 NSCH. Missing values for child demographic variables were imputed using hot-deck imputation and the adult education, household size, and poverty ratio missing values were imputed using sequential regression imputation methods [29,30,31].

Physical activity, the dependent variable, was one item on the NSCH that asked, “During the past week, on how many days did this child exercise, play a sport, or participate in physical activity for at least 60 min?” Reponses for this item ranged from 0 days to every day. Because child and adolescent physical activity guidelines indicate participating in at least 60 min of physical activity per day, a binary dependent variable was derived categorizing children that met the 60 min per day guideline (coded 1) and children who did not meet the 60 min per day guideline (coded 0). A primary predictor variable was a composite measure on the NSCH that assessed family resilience. This composite measure consisted of four items asking the parents “When your family faces problems, how often are you likely to do each of the following?” (a) Talk together about what to do, (b) Work together to solve our problems, (c) Know we have strengths to draw on, and (d) Stay hopeful even in difficult times. Scale reliability for the family resilience composite was Cronbach’s alpha = 0.89. The family resilience composite variable was then categorized into three levels indicating children who live in families that met 0–1 family resilience items, children who live in families that met 2–3 resilience items, and children who live in families that met all 4 resilience items. Another primary predictor variable was the event attendance item that asked, “During the past 12 months, how often did you (the parent) attend events or activities that this child participated in?” Responses ranged from rarely, sometimes, usually, and always. Because of the small prevalence of “rarely” and “sometimes” responses, the event attendance variable was dichotomized into rarely/sometimes and usually/always for analysis.

Other categorical family predictor variables included items regarding mother and father rated health (poor/fair/good, very good/excellent), generational status (all parents born in the US, any parent born outside of the US), highest adult education within household (college degree or higher, some college, high school/General Educational Development (GED), less than high school), family income (400% federal poverty level (FPL) or greater, 200–399% FPL, 100–199% FPL, 0–99% FPL), and the family structure (two married parents, two un-married parents, single parent, grandparent). Child-level covariates were also included in the analysis and consisted of child age (6–11 years, 12–17 years), child sex (female, male), child body mass index (BMI) class (not overweight or obese, overweight or obese), and child race/ethnicity (White-Non-Hispanic, Hispanic, Black-Non-Hispanic, Other/Multi-Racial-Non-Hispanic). The BMI class variable was sex-specific BMI-for-age categories calculated using the child’s age in months and the sex-specific 2000 Centers for Disease and Prevention Growth Charts [29].

### 2.4. Statistical Analysis

To account for NSCH’s complex survey design and sampling weight, the STATA survey prefix command “svy” was employed to compute weighted prevalence within the descriptive analysis and to compute appropriate variances and confidence intervals within the primary analysis. Unweighted and weighted prevalence statistics were computed for all physical activity and familial variables. Because the physical activity dependent variable was on the categorical measurement scale and binary coded, the primary analysis consisted of the use of weighted logistic regression models. Crude parameter estimates were calculated for all predictors to assess associations with child physical activity. Predictor variables were then entered into the model using hierarchical (block-wise) entry. Model 1 consisted of regressing child physical activity on all familial predictor variables. Model 2 consisted of regressing child physical activity on all familial predictors and child-level covariates. Communication of the results involved the reporting of odds ratios (ORs) with the associated 95% confidence intervals (CI). Marginal predicted probability plots were also constructed to display predicted probabilities of meeting the various weekly frequencies of 60 min of daily physical activity for predictors showing relatively strong associations with child physical activity. Because the social ecological model assumes interaction between levels, secondary analyses were conducted to test for joint associations (interactions) between family-level and child-level variables. Each family-level variable and child-level variable were tested using 2-way interactions within weighted logistic regression models to examine the joint effects on a child meeting the 60 min of physical activity per day guideline. Statistically significant joints effects were communicated within text and in graphical format. The two-sided alpha level was set at *p* < 0.05 and all analyses were conducted using STATA v15.0 statistical software package (StataCorp, College Station, TX, USA).

## 3. Results

### 3.1. Child-Level Demographics

Adolescents aged 12–17 years old comprised of 57.5% of the sample (*n* = 21,496) and children aged 6–11 years old comprised of 42.5% of the sample (*n* = 15,896). The sample was 48.8% female (*n* = 17,977) and 51.2% male (*n* = 19,415). Approximately 27.6% of the sample was overweight or obese (*n* = 7255) with 6.5% of the sample being underweight (*n* = 1701). Non-Hispanic Whites comprised the majority of the sample (69.2%; *n* = 25,867), followed by Hispanic Whites (11.7%, *n* = 4377), Non-Hispanic Blacks (6.7%; *n* = 2497), and 12.4% (*n* = 4651) identified as “Other/Multi-Racial”.

### 3.2. Physical Activity and Family-Level Descriptive Statistics

Descriptive statistics for child physical activity and the familial factors, including raw counts and unweighted and weighted prevalence, are reported in Table 1. The unweighted prevalence of children reported to have engaged in 60 min of physical activity everyday was 21.0% with a weighted prevalence of 22.6%. Familial factors with the highest prevalence included meeting all four family resilience items, usually/always event attendance, very good/excellent mother’s and father’s health, being born in the US, a college degree or higher adult education, a family income of 400% FPL or higher, and a two-married parent family structure.

### 3.3. Weighted Logistic Regression Results

**Figure 1:**
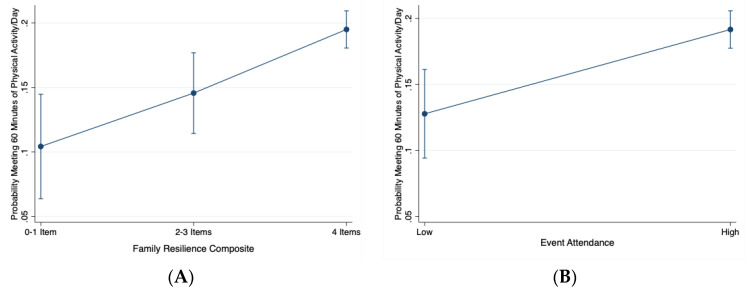
Marginal predicted probabilities of meeting physical activity guidelines, stratified by levels of family resilience (**A**—**Left**) and event attendance (**B**—**Right**). Note: Low is Rarely or Sometimes event attendance; High is Usually or Always event attendance for Figure 1B. Probabilities are adjusted for other familial and child-level covariates.

Table 2 reports the results of the weighted logistic regression models. The highest level of family resilience (meeting all four family resilience items) significantly associated with a child meeting the 60 min of physical activity per day guideline (OR = 1.80, 95%CI: 1.28–2.53, *p* = 0.001; Model 1). This relationship held after further adjustment for pertinent child-level covariates (OR = 2.17; 95%CI: 1.35–3.49, *p* = 0.001; Model 2). Additionally, parents usually or often attending their child’s activities or events significantly associated with a child meeting the 60 min of physical activity per day guideline compared to parents who rarely or sometimes attended their child’s events (OR = 1.72; 95%CI: 1.29–2.29; *p* < 0.001; Model 1). This relationship held after further adjustment for pertinent child-level covariates (OR = 1.65; 95%CI: 1.18–2.31, *p* = 0.004; Model 2). Figure 1A,B show the marginal predicted probabilities of a child meeting 60 min of physical activity per day guideline for the family resilience and event attendance predictor variables, respectively. The only other family-level factor associating with child physical activity was family income. In the fully adjusted model (Model 2), having a family income of 200–399% federal poverty level (FPL) associated with higher odds of a child meeting the 60 min of physical activity per day guideline compared to having a poverty level of 0–99% FPL (OR = 1.60, 95%CI: 1.24–2.04, *p* < 0.001). Likewise, having a family income of 400% FPL or greater associated with higher odds of a child meeting the 60 min of physical activity per day guideline compared to having a poverty level of 0–99% FPL (OR = 1.84, 95%CI: 1.34–2.52, *p* < 0.001).

Child-level covariates that significantly associated with lower reported child weekly physical activity included ages 12–17 years old compared to ages 6–11 years old (OR = 0.63; 95%CI: 0.52–0.78, *p* < 0.001), female children compared to male children (OR = 0.56; 95%CI: 0.56–0.65, *p* < 0.001), and overweight or obese children compared to non-overweight or obese children (OR = 0.49; 95%CI: 0.39–0.61, *p* < 0.001). Child race/ethnicity did not significantly associate with meeting the 60 min of physical activity per day guideline within the fully adjusted model.

### 3.4. Family-Level and Child-Level Joint Associations

**Figure 2:**
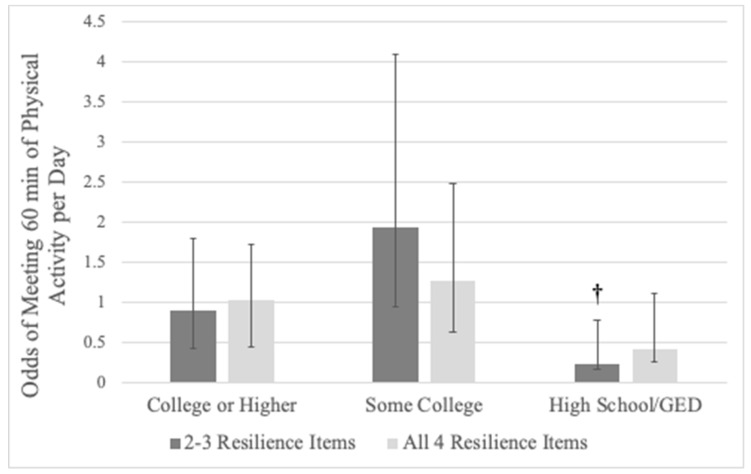
Joint association of adult education and family resilience on the odds of a child meeting the 60 min of physical activity per day guideline. Note: Referent for adult education is lower than high school; referent for family resilience is meeting 0–1 items; error bars are 95% CIs; † denotes a statistically significant association compared to referent levels, *p* < 0.05.

Families with a member being born outside of the US and meeting 2–3 items of family resilience associated with lower odds of a child meeting the 60 min of physical activity per day guideline (OR = 0.41, 95%CI: 0.17–0.99, *p* = 0.048). Additionally, families with a highest adult education of a high school diploma/GED and meeting 2–3 items of family resilience associated with lower odds of a child meeting the 60 min of physical activity per day guideline (OR = 0.23, 95%CI: 0.07–0.75, *p* = 0.015). Figure 2 depicts the joint association between family resilience and adult education with child physical activity. No other statistically significant joint associations were observed between any other familial and child-level variable on the odds of a child meeting the 60 min of physical activity per day guideline.

## 4. Discussion

The purpose of this study was to examine the associations between familial factors and weekly child physical activity within a representative sample of US children and adolescents using data from the combined 2017–2018 NSCH. The results indicated that higher levels of familial resilience, high parental attendance during child events or activities, and high family income was associated with higher child physical activity. Family generational status and adult education significantly modified the association between family resilience and child physical activity. Our findings suggest that interventions for promoting physical activity in children and adolescents should incorporate family-level components. An interpretation of the findings and a discussion of the public health implications are provided further.

A primary finding from the current study was that higher levels of familial resilience correlated with higher odds that a child met the 60 min of physical activity per day guideline. To the authors’ knowledge, this is the first study to examine the correlation specifically between family resilience and youth physical activity. This association was modified by family generational status and adult education levels. Previous work has revealed positive associations between higher levels of family resilience and psychosocial variables such as parental social support [23,24]. Higher levels of family resilience also associate with higher child resilience [32], which in turn correlates with positive health behaviors [25,26]. The current study’s results suggest there may be a direct link between family resilience and child physical activity. Although family generational status only slightly modified this association, having a family member being born outside of the US may bring into the family unit different cultural norms that may modify child physical activity behaviors and weaken the association between resilience and physical activity [33]. Another effect modifier in this relationship was adult education. Adult education has shown to negatively correlate with child physical activity in other work [34]. Lower levels of adult education may weaken the association between family resilience and child physical activity. Adults with lower levels of education may not have the resources needed to provide support for children for the promotion of physical activity or may not be knowledgeable of the benefits of physical activity for their children. These complex interrelationships are recommended to be explored in future research using more rigorous research methodologies.

Another primary finding from the analysis was that parents who usually or always attend their child’s events or activities significantly correlated with higher reported child physical activity; this finding was consistent with previous findings that parental social support is an important construct for child health behaviors [35]. Previous work has shown that higher levels of perceived parental social support for physical activity also correlates with improvements in other psychosocial factors within children such as physical activity enjoyment and self-efficacy and can lead to sustained physical activity [36,37]. Creating a supporting environment has been shown to manifest higher levels of health behaviors within physical activity intervention studies [38]. Many school- and community-based interventions have recommended familial components to their work [16], however, these components tend to be difficult to implement because of scheduling and time constraints of the parent [16,39]. Given the results from the current study, interventions using a family support component may want to convey messages that supporting their child in their endeavors, for instance simply by attending their events, may foster a home environment that may correlate to improved levels of physical activity. Because of the broadness of the item linked to parental support in the current study, it is unknown if parents attending their child’s events were indeed activity-based.

Although a link between event attendance and physical activity was observed in the current study, event attendance is only one type of parental social support [40]. Davison et al. [41], using the Activity Support Scale, examined the association between parental support and adolescent physical activity by combining two types of support, “logistical” (making arrangements so a child can be active) and “modeling” (doing activities with children). Results indicated that activity-related support from all sources associated with higher levels of physical activity in adolescents, with a stronger association observed in adolescents at high risk for physical inactivity. Beets et al. [28] categorized parental social support into two broad categories of “tangible” (overt behaviors to facilitate physical activity) and “intangible” (verbal encouragement) social support. “Tangible” social support was further categorized to “instrumental transportation”, “instrumental-purchasing equipment”, “conditional-perform activity together”, and “conditional-watching/supervision”. “Logistical” social support, as defined by Davison et al. [41] and “conditional-watching supervision” as defined by Beets et al. [28] align with the type of social support examined in the current study that is event attendance. Like results seen in past research, conditional-watching/supervision support (such as event attendance) correlates with child physical activity behaviors [42,43,44]. This association in the current study was robust, as the relationship held even after controlling for other familial and child-level covariates. However, this association, unlike those found in Davison et al. [41], was not modified by any other familial or child-level factor, suggesting that this determinant of child physical activity is independent of other familial or child-level determinants. Nevertheless, given the results of past studies and the current study, providing parental education on the importance of social support for the promotion of child physical activity may be an important consideration for intervention programs. Because of the various types of social support, educating parents on these different ways to provide support for their children, whether it is “logistical/conditional-watching/supervision” by watching a child’s events or activities or “modeling/conditional-perform activity together” by engaging in parent–child activities together or performing activities together as a larger family unit, may enhance school and community-based physical activity programming.

The only other familial factor that significantly related with child physical activity was family income level. In the current study, higher levels of family income associated with higher odds of a child meeting the 60 min of physical activity per day guideline. Lower family income has been shown to negatively influence child health behaviors, such as physical activity, and subsequent weight status [45]. Within the current study, after statistical adjustment for a number of family-level and child-level covariates, higher family income was a robust determinant of child physical activity. This finding agrees with a number of previous research studies linking family income with youth physical activity and reinforces the need to develop physical activity interventions for children who are from lower income families [46,47]. Although, these efforts have been utilized recently, infusing parental education and family components into these physical activity programs is a challenge because of the barriers within lower income families that may potentially mitigate child physical activity promotion efforts [48,49]. Child-level covariates showing negative correlation with child physical activity included the older age group (12–17 years old) compared to the younger age group (6–11 years old), being female compared to male, and being overweight/obese compared to not being overweight/obese. Previous research has consistently found these correlations in past research and, thus, the significant associations between the child-level covariates with reported weekly child physical activity was expected [50].

This study has several strengths to highlight. First, this study included a large and representative sample of US children and adolescents, thus the generalizability of findings from this study is not limited. Second, this study included a wide range of familial factors measured from a population-based survey. Lastly, the analyses conducted in this study included the combined data from two consecutive years of the NSCH, which improves statistical power and provides precise parameter estimates compared to one-year data. Limitations of the current study include the use of parent self-report, which may be subject to both recall and response bias. The NSCH is an annual cross-sectional survey, therefore all relationships are correlational and no causal inferences can be made. The direction of the association is also questionable, as the link between parental support and child physical activity may be bidirectional. Additionally, the physical activity-dependent variable only asked about meeting the weekly frequency of the 60-min per day guideline but did not specifically ask about physical activity intensity. The physical activity outcome may be sensitive to social desirability and overestimation. Finally, the event attendance variable asked about attendance to child events but did not ask about specific types of events or more specific behaviors relative to the domain of child physical activity (e.g., sports attendance transportation, sports team volunteer, physical activity encouragement, perceptions of physical activity engagement). Furthermore, the event attendance variable was skewed even after merging the original categories for analysis; therefore, results should be interpreted with caution.

## 5. Conclusions

The results of this analysis suggest that parental support in their child’s events may be an important factor in promoting physical activity. Parental characteristics and behaviors correlate with child physical activity, even after adjustment for important child-level factors. Public health messages regarding the parents’ important role in the promotion of physical activity may not be as clear or as strong as they could be to improve child movement behaviors. The current study provides robust evidence of family-level determinants of child physical activity. These findings should spur additional research using more rigorous research designs and objective measures to improve internal validity evidence. Nevertheless, because of current study’s findings, parents may have as much of a role and responsibility to promote child physical activity as anyone in a child’s life and, thus, improving family resilience and involving parental support within school- and community-based interventions to promote child physical activity should be considered.

## Figures and Tables

**Table 1:** Descriptive statistics for child physical activity and familial predictor variables.

Variable	Level	Count	Unweighted %	Weighted %
Child Physical Activity	Not 60 min/day	29,213	79.0%	77.4%
	60 min/day	7767	21.0%	22.6%
Family Resilience	0–1 item	2474	6.7%	7.4%
	2–3 items	4305	11.7%	12.1%
	4 items	30,130	81.6%	80.5%
Event Attendance	Rarely/Sometimes	3762	10.2%	14.6%
	Usually/Always	33,141	89.8%	85.4%
Mother’s Health	Poor/Fair/Good	9498	28.7%	30.9%
	Very Good/Excellent	23,584	71.3%	69.1%
Father’s Health	Poor/Fair/Good	8017	28.0%	28.8%
	Very Good/Excellent	20,615	72.0%	71.2%
Generational Status	Born in US	28,729	77.7%	65.5%
	Born Outside of US	8244	22.3%	34.5%
Adult Education	College Degree or Higher	22,195	59.4%	47.3%
	Some College	9063	24.3%	22.3%
	High School/GED	5127	13.7%	19.8%
	Less than high school	1007	2.7%	10.6%
Family Income	0–99% FPL	4566	12.2%	20.2%
	100–199% FPL	5884	15.7%	21.7%
	200–399% FPL	11,248	30.8%	27.1%
	400% FPL or greater	15,694	42.0%	31.0%
Family Structure	Two Married Parents	25,744	70.3%	64.7%
	Two Un-Married Parents	2239	6.1%	8.0%
	Single Parent	7108	19.3%	21.3%

Note: GED stands for General Educational Development; FPL stands for federal poverty level.

**Table 2:** Parameter estimates from the weighted logistic regression model on parent-reported child physical activity.

Predictor	Level	Crude OR (95% CI)	Model 1 aOR (95% CI)	Model 2 aOR (95% CI)
Family Resilience	0–1 Items	referent	referent	referent
	2–3 items	**1.29** ^**†**^ **(0.90–1.84)**	1.30 (0.89–1.91)	1.55 (0.92–2.65)
	4 items	**1.70** ^**†**^ **(1.23–2.35)**	**1.80** ^**†**^ **(1.28–2.53)**	**2.17** ^**†**^ **(1.35–3.49)**
Event Attendance	Rarely/Sometimes	referent	referent	referent
	Usually/Always	**1.31** ^**†**^ **(1.14–1.70)**	**1.72** ^**†**^ **(1.29–2.29)**	**1.65** ^**†**^ **(1.18–2.31)**
Mother’s Health	Poor/Fair/Good	referent	referent	referent
	Very Good/Excellent	**1.16** ^**†**^ **(1.01–1.32)**	1.17 (0.99–1.40)	1.15 (0.92–1.45)
Father’s Health	Poor/Fair/Good	referent	referent	referent
	Very Good/Excellent	**1.22** ^**†**^ **(1.06–1.41)**	1.13 (0.96–1.33)	1.01 (0.81–1.25)
Generational Status	Born in US	referent	referent	referent
	Born Outside US	0.90 (0.82–1.00)	**0.72** ^**†**^ **(0.60–0.86)**	0.81 (0.62–1.05)
Adult Education	College Degree	referent	referent	referent
	Some College	0.93 (0.85–1.02)	1.11 (0.93–1.32)	1.24 (0.99–1.49)
	High School/GED	**0.79** ^**†**^ **(0.70–0.90)**	1.11 (0.89–1.39)	0.95 (0.78–1.16)
	Less than high school	**0.57** ^**†**^ **(0.45–0.72)**	1.06 (0.71–1.61)	0.87 (0.58–1.32)
Family Income	0–99% FPL	referent	referent	referent
	100–199% FPL	1.10 (0.86–1.43)	**1.15** ^**†**^ **(1.00–1.33)**	1.17 (0.98–1.41)
	200–399% FPL	**1.22** ^**†**^ **(1.05–1.42)**	**1.57** ^**†**^ **(1.29–1.91)**	**1.60** ^**†**^ **(1.24–2.04)**
	400% FPL Greater	**1.22** ^**†**^ **(1.07–1.38)**	**1.71** ^**†**^ **(1.31–2.25)**	**1.84** ^**†**^ **(1.34–2.52)**
Family Structure	Two Married Parents	referent	referent	referent
	Two Un-Married Parents	1.21 (0.96–1.53)	1.27 (0.8–1.64)	1.50 (0.97–2.16)
	Single Parent	1.05 (0.92–1.21)	0.63 (0.13–2.90)	0.67 (0.09–4.62)
	Grandparent Household	1.38 (1.06–1.80)	0.46 (0.17–1.21)	0.35 (0.08–1.70)
Child Age	6–11 years old	referent	referent	referent
	12–17 years old	**0.55** ^**†**^ **(0.49–0.62)**		**0.63** ^**†**^ **(0.52–0.78)**
Child Sex	Male	referent	referent	referent
	Female	**0.68** ^**†**^ **(0.62–0.76)**		**0.56** ^**†**^ **(0.46–0.65)**
Child BMI Class	Not Overweight/Obese	referent	referent	referent
	Overweight/Obese	**0.82** ^**†**^ **(0.75–0.89)**		**0.49** ^**†**^ **(0.39–0.61)**
Child Race/Ethnicity	White/Non-Hispanic	referent	referent	referent
	Hispanic	0.85 (0.72–1.01)		0.90 (0.65–1.23)
	Black, Non-Hispanic	0.92 (0.78–1.08)		0.86 (0.62–1.19)
	Other/Multi-racial	**0.81** ^**†**^ **(0.70–0.94)**		0.90 (0.68–1.20)

Note: Model 1 is familial predictors only; Model 2 is familial predictors and child-level covariates; OR stands for odds ratio; aOR stands for adjusted odds ratio; 95% CI stands for 95% confidence interval; FPL stands for federal poverty level; BMI stands for body mass index; bold and ^†^ denotes statistical significance, *p* < 0.05.

## References

[1] Ekelund U., Luan J., Sherar L.B., Eslinger D.W., Griew P., Cooper A., International Children’s Accelerometry Database (ICAD) Collaborators (2012). Moderate to vigorous physical activity and sedentary time and cardio-metabolic risk factors in children and adolescents. JAMA.

[2] Hillman C.H., Erickson K.I., Kramer A.F. (2008). Be smart, exercise your heart: Exercise effects on brain and cognition. Nat. Rev. Neurosci..

[3] Biddle S.J., Asare M. (2011). Physical activity and mental health in children and adolescents: A review of reviews. Br. J. Sports Med..

[4] National Physical Activity Plan Alliance (2018). The 2018 United States Report Card on Physical Activity for Children and Youth.

[5] Guthold R., Stevens G.A., Riley L.M., Bull F.C. (2020). Global trends in insufficient physical activity among adolescents: A pooled analysis of 298 population-based surveys with 1.6 million participants. Lancet Child. Adolesc. Health.

[6] Pate R.R., Dowda M., Dishman R.K., Colabianchi N., Saunders R.P., McIver K.L. (2019). Change in children’s physical activity: Predictors in the transition from elementary to middle school. Am. J. Prev. Med..

[7] Rebold M.J., Lepp A., Kobak M.S., McDaniel J., Barkley J.E. (2016). The effect of parental involvement on children’s physical activity. J. Pediatr..

[8] Zecevic C.A., Tremblay L., Lovsin T., Michel L. (2010). Parental influence on young children’s physical activity. Int. J. Pediatr..

[9] Gapin J.I., Etnier J.L. (2014). Parental perceptions of the effects of exercise on behavior in children and adolescents with ADHD. J. Sport Health Sci..

[10] Solomon-Moore E., Toumpakari Z., Sebire S.J., Thompson J.L., Lawlor D.A., Jago R. (2018). Roles of mothers and fathers in supporting child physical activity: A cross-sectional mixed-methods study. BMJ Open.

[11] Sallis J.F., Cervero R.B., Ascher W., Henderson K.A., Kraft M.K., Kerr J. (2006). An ecological approach to creating active living communities. Ann. Rev. Public Health.

[12] Burns R.D., Bai Y., Fu Y., Pfledderer C.D., Brusseau T.A. (2019). Parent engagement and support, physical activity and academic performance (PESPAAP): A proposed theoretical model. Int. J. Env. Res. Public Health.

[13] Brown H.E., Atkin A.J., Panter J., Wong G., Chinapaw M.J.M., van Sluijs E.M.F. (2016). Family-based interventions to increase physical activity in children: A systematic review, meta-analysis, and realist synthesis. Obes. Rev..

[14] Burns R.D. (2019). Enjoyment, self-efficacy, and physical activity within parent-adolescent dyads: Application of the Actor-Partner Interdependence Model. Prev. Med..

[15] Davison K.K., Cutting T.M., Birch L.L. (2003). Parents’ activity-related parenting practices predict girls’ physical activity. Med. Sci. Sports Exerc..

[16] Mutz M., Albrecht P. (2017). Parents’ social status and children’s daily physical activity: The role of familial socialization and support. J. Child Fam. Stud..

[17] Maher J.P., Ra C., O’Connor S.G., Belcher B.R., Leventhal A., Margolin G., Dunton G.F. (2017). Associations between maternal mental health and well-being and physical activity and sedentary behavior in children. J. Dev. Behav. Pediatr..

[18] Liu J., Probst J.C., Harun N., Bennett K.J., Torres M.E. (2009). Acculturation, physical activity, and obesity among Hispanic adolescents. Ethn. Health.

[19] Muthuri S.K., Onywera V.O., Tremblay M.S., Broyles S.T., Chaput J.P., Fogelholm M., Hu G., Kuriyan R., Kurpad A., Lambert E.V. (2016). Relationships between parental education and overweight with childhood overweight and physical activity in 9-11 year old children: Results from a 12 country study. PLoS ONE.

[20] Min J., Xue H., Wang Y. (2018). Association between household poverty dynamics and childhood overweight risk and health behaviours in the United States: A 8-year nationally representative longitudinal study of 16 800 children. Pediatr. Obes..

[21] Langoy A., Smith O.R.F., Wold B., Samdal O., Hang E.M. (2019). Associations between family structure and young people’s physical activity and screen time behaviors. BMC Public Health.

[22] Walsh F. (2016). Family resilience: A developmental systems framework. Eur. J. Dev. Psychol..

[23] Bethell C.D., Gombojav N., Whitaker R.C. (2019). Family resilience and connection promote flourishing among US children, even amid adversity. Health Aff..

[24] Armstrong M.I., Birnie-Lefcovitch S., Ungar M.T. (2005). Pathways between social support, family well-being, quality of parenting, and child resilience: What we know. J. Child. Fam. Stud..

[25] Jeffries P., Ungar M., Aubertin P., Kriellaars D. (2019). Physical literacy and resilience in children and youth. Front. Public Health.

[26] Ho F.K., Louie L.H., Chow C.B., Wong W.H., Ip P. (2015). Physical activity improves mental health through resilience in Hong Kong Chinese adolescents. BMC Pediatr..

[27] Morgan P.J., Young M.D., Barnes A.T., Eather N., Pollock E.R., Lubans D.R. (2019). Engaging father to increase activity in girls: The “Dads and Daughters Exercising and Empowered (DADEE) Randomized Controlled Trial. Ann. Behav. Med..

[28] Beets M.W., Cardinal B.J., Alderman B.L. (2010). Parental social support and the physical activity-related behaviors of youth: A review. Health Educ. Behav..

[29] Child and Adolescent Health Measurement Initiative (CAHMI) (2019) 2017–2018 National Survey of Children’s Health (2 Years Combined Data Set): Child and Family Health Measures, National Performance and Outcome Measures, and Subgroups, STATA Codebook, Version 1.0, Data Resource Center for Child and Adolescent Health supported by Cooperative Agreement U59MC27866 from the U.S. Department of Health and Human Services, Health Resources and Services Administration (HRSA), Maternal and Child Health Bureau (MCHB). https://www.childhealthdata.org.

[30] Child and Adolescent Health Measurement Initiative 2018 National Survey of Children’s Health, Sampling and Survey Administration. Data Resource Center for Child and Adolescent Health, Supported by Cooperative Agreement 1-U59-MC06980-01 from the U.S. Department of Health and Human Services, Health Resources and Services Administration (HRSA), Maternal and Child Health Bureau (MCHB). https//www.childhealthdata.org.

[31] The United States Census Bureau Associate Director of Demographic Programs, National Survey of Children’s Health. 2018 National Survey of Children’s Health Frequently Asked Questions. September 2019. https://www2.census.gov/programs-surveys/nsch/technical-documentation/methodology/2018-NSCH-FAQs.pdf.

[32] Coyle J. (2011). Resilient families help make resilient children. J. Fam. Strengths.

[33] Jaeschke L., Steinbrecher A., Luzak A., Puggina A., Aleksovska K., Buck C., Burns C., Cardon G., Carlin A., Chantal S. (2017). Socio-cultural determinants of physical activity across the life course: A ‘Determinants of Diet and Physical Activity’ (DEDIPAC) umbrella systematic literature review. Int. J. Behav. Nutr. Phys. Act..

[34] McMinn A.M., Griffin S.J., Jones A.P., van Sluijs E.M.F. (2013). Family and home influences on children’s after-school and weekend physical activity. Eur. J. Public Health.

[35] Pyper E., Harrington D., Manson H. (2016). The impact of different types of parental support behaviours on child physical activity, healthy eating, and screen time: A cross-sectional study. BMC Public Health.

[36] Dowda M., Dishman R.K., Pfeiffer K.A., Pate R.R. (2007). Family support for physical activity in girls from 8th to 12th grade in South Carolina. Prev. Med..

[37] Dishman R.K., Motl R.W., Saunders R., Felton G., Ward D.S., Dowda M., Pate R.R. (2005). Enjoyment mediates effects of a school-based physical-activity intervention. Med. Sci. Sports Exerc..

[38] Eather N., Morgan P.J., Lubans D.R. (2013). Social support from teacher mediates physical activity behavior change in children participating in the Fit-4-Fun intervention. Int. J. Behav. Nutr. Phys. Act..

[39] Bentley G.F., Goodred J.K., Jago R., Sebire S.J., Lucas P.J., Fox K.R., Stewart-Brown S., Turner K.M. (2012). Parents’ views on child physical activity and their implications for physical activity parenting interventions: A qualitative study. BMC Pediatr..

[40] Laird Y., Fawkner S., Kelly P., McNamee L., Niven A. (2016). The role of social support on physical activity behavior in adolescent girls: A systematic review and meta-analysis. Int. J. Behav. Nutr. Phys. Act..

[41] Davison K.K., Schmalz D.L. (2006). Youth at risk of physical inactivity may benefit more from activity-related support than youth not at risk. Int. J. Behav. Nutr. Phys. Act..

[42] Duncan S.C., Duncan T.E., Strycker L.A. (2005). Sources and type of social support in youth physical activity. Health Psychol..

[43] Heitzler C.D., Martin S.L., Duke J., Huhman M. (2006). Correlates of physical activity in a national sample of children aged 9–13 years. Prev. Med..

[44] Prochaska J.J., Rodgers M.W., Sallis J.F. (2002). Association of parent and peer support with adolescent physical activity. Res. Q Exerc. Sport.

[45] Anselma M., Chinapaw M.J.M., Altenburg T.M. (2018). Determinants of child health behaviors in a disadvantaged area from a community perspective: A participatory needs assessment. Int. J. Env. Res. Public Health.

[46] Kantomaa M.T., Tammelin T.H., Nayha S., Taanila A.N. (2007). Adolescents physical activity in relation to family income and parental education. Prev. Med..

[47] Duncan S.C., Strycker L.A., Chaumeton N.R. (2015). Personal, family, and peer correlates of general and sport physical activity among African American, Latino, and White Girls. J. Health Dispar. Res. Pr..

[48] Romero A.J. (2005). Low-income neighborhood barriers and resources for adolescents’ physical activity. J. Adolesc. Health.

[49] Gordon-Larsen P., Nelson M.C., Page P., Popkin B.M. (2006). Inequality in the built environment underlies key health disparities in physical activity and obesity. Pediatrics.

[50] Stanley R.M., Ridley K., Dollman J. (2012). Correlates of children’s time-specific physical activity: A review of the literature. Int. J. Behav. Nutr. Phys. Act..

